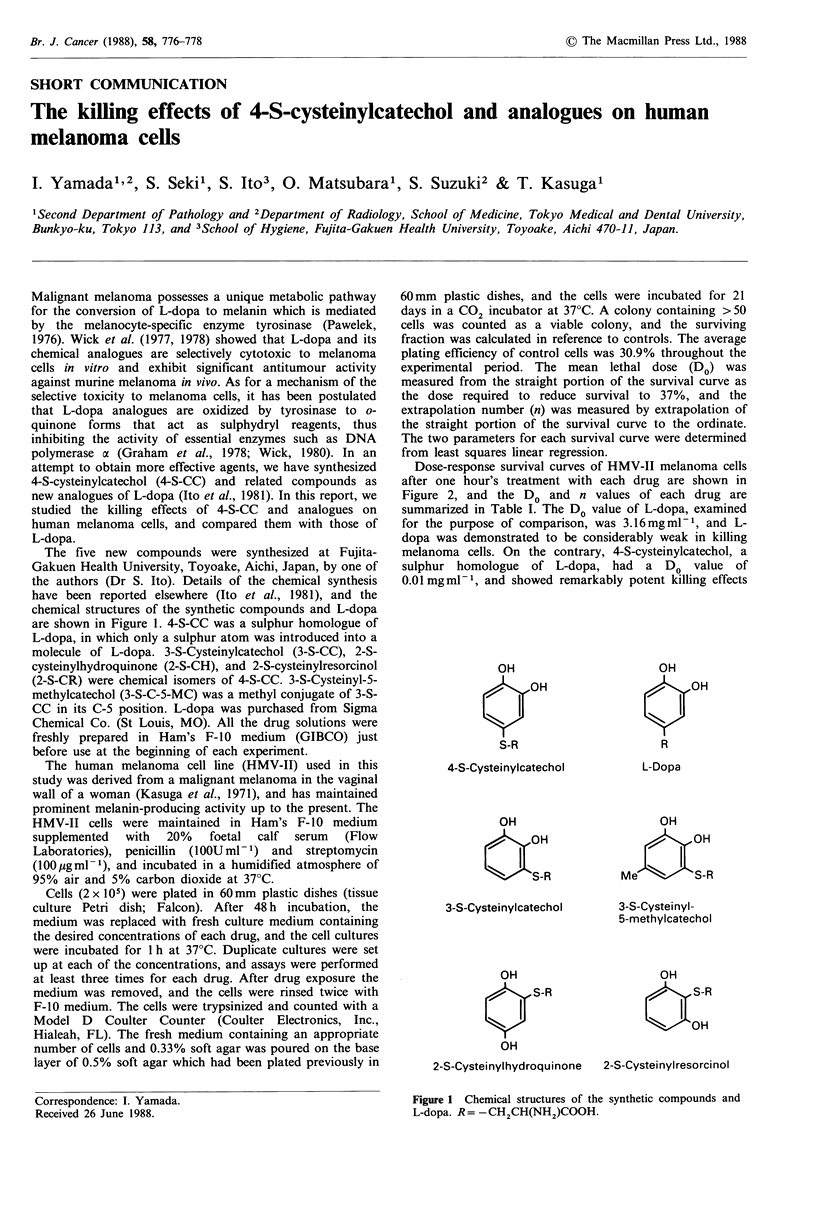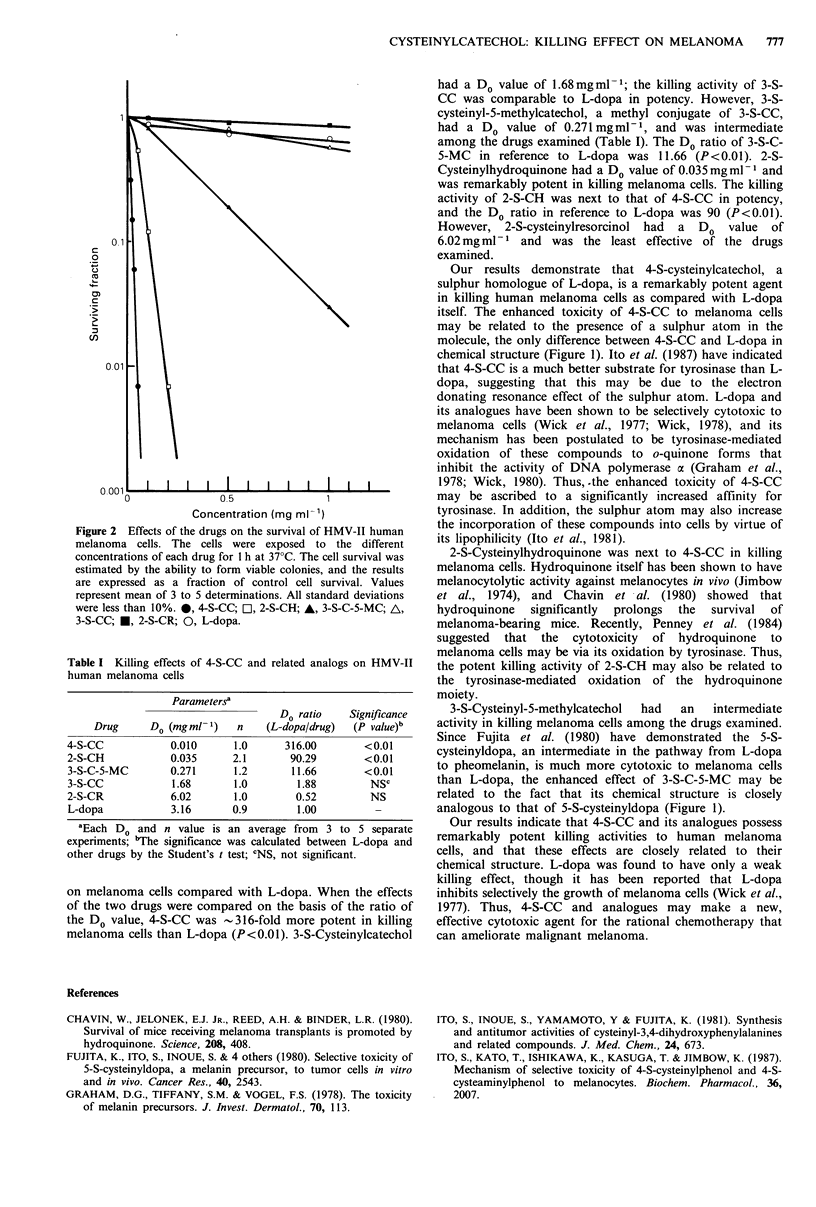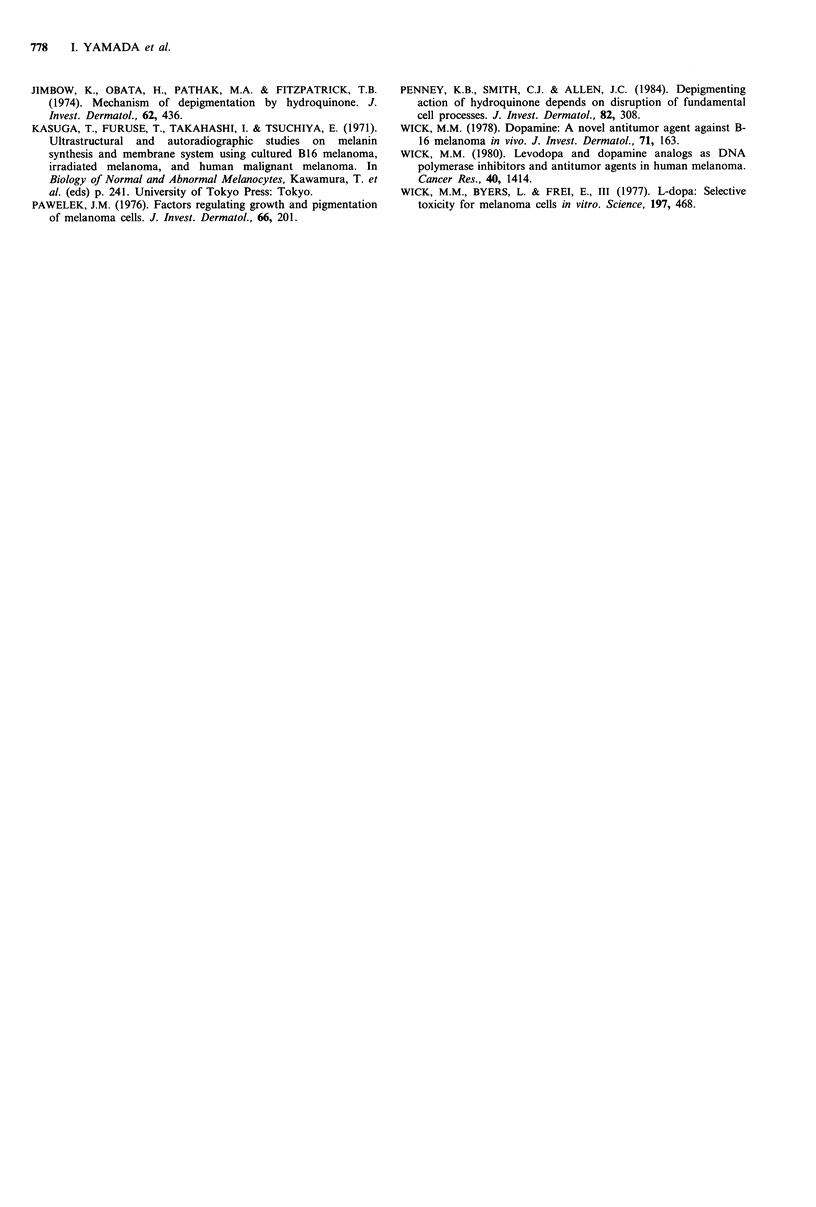# The killing effects of 4-S-cysteinylcatechol and analogues on human melanoma cells.

**DOI:** 10.1038/bjc.1988.307

**Published:** 1988-12

**Authors:** I. Yamada, S. Seki, S. Ito, O. Matsubara, S. Suzuki, T. Kasuga

**Affiliations:** Second Department of Pathology, School of Medicine, Tokyo Medical and Dental University, Japan.


*Br. J. Cancer* (1988), **58**, 776–778 © The Macmillan Press Ltd., 1988

SHORT COMMUNICATION

### The killing effects of 4-S-cysteinylcatechol and analogues on human melanoma cells

I. Yamada[1,2], S. Seki[1], S. Ito[3], O. Matsubara[1], S. Suzuki[2] & T. Kasuga[1]

[1]*Second Department of Pathology and* [2]*Department of Radiology, School of Medicine, Tokyo Medical and Dental University, Bunkyo-ku, Tokyo 113, and* [3]*School of Hygiene, Fujita-Gakuen Health University, Toyoake, Aichi 470-11, Japan.*

Malignant melanoma possesses a unique metabolic pathway for the conversion of L-dopa to melanin which is mediated by the melanocyte-specific enzyme tyrosinase (Pawelek, 1976). Wick *et al.* (1977, 1978) showed that L-dopa and its chemical analogues are selectively cytotoxic to melanoma cells *in vitro* and exhibit significant antitumour activity against murine melanoma *in vivo*. As for a mechanism of the selective toxicity to melanoma cells, it has been postulated that L-dopa analogues are oxidized by tyrosinase to *o*-quinone forms that act as sulphydryl reagents, thus inhibiting the activity of essential enzymes such as DNA polymerase α (Graham *et al.*, 1978; Wick, 1980). In an attempt to obtain more effective agents, we have synthesized 4-S-cysteinylcatechol (4-S-CC) and related compounds as new analogues of L-dopa (Ito *et al.*, 1981). In this report, we studied the killing effects of 4-S-CC and analogues on human melanoma cells, and compared them with those of L-dopa.

The five new compounds were synthesized at Fujita-Gakuen Health University, Toyoake, Aichi, Japan, by one of the authors (Dr S. Ito). Details of the chemical synthesis have been reported elsewhere (Ito *et al.*, 1981), and the chemical structures of the synthetic compounds and L-dopa are shown in Figure 1. 4-S-CC was a sulphur homologue of L-dopa, in which only a sulphur atom was introduced into a molecule of L-dopa. 3-S-Cysteinylcatechol (3-S-CC), 2-S-cysteinylhydroquinone (2-S-CH), and 2-S-cysteinylresorcinol (2-S-CR) were chemical isomers of 4-S-CC. 3-S-Cysteinyl-5-methylcatechol (3-S-C-5-MC) was a methyl conjugate of 3-S-CC in its C-5 position. L-dopa was purchased from Sigma Chemical Co. (St Louis, MO). All the drug solutions were freshly prepared in Ham's F-10 medium (GIBCO) just before use at the beginning of each experiment.

The human melanoma cell line (HMV-II) used in this study was derived from a malignant melanoma in the vaginal wall of a woman (Kasuga *et al.*, 1971), and has maintained prominent melanin-producing activity up to the present. The HMV-II cells were maintained in Ham's F-10 medium supplemented with 20% foetal calf serum (Flow Laboratories), penicillin ($100 U ml^{-1}$) and streptomycin ($100 \mu g ml^{-1}$), and incubated in a humidified atmosphere of 95% air and 5% carbon dioxide at 37°C.

Cells ($2 \times 10^5$) were plated in 60 mm plastic dishes (tissue culture Petri dish; Falcon). After 48 h incubation, the medium was replaced with fresh culture medium containing the desired concentrations of each drug, and the cell cultures were incubated for 1 h at 37°C. Duplicate cultures were set up at each of the concentrations, and assays were performed at least three times for each drug. After drug exposure the medium was removed, and the cells were rinsed twice with F-10 medium. The cells were trypsinized and counted with a Model D Coulter Counter (Coulter Electronics, Inc., Hialeah, FL). The fresh medium containing an appropriate number of cells and 0.33% soft agar was poured on the base layer of 0.5% soft agar which had been plated previously in 60 mm plastic dishes, and the cells were incubated for 21 days in a $CO_2$ incubator at 37°C. A colony containing >50 cells was counted as a viable colony, and the surviving fraction was calculated in reference to controls. The average plating efficiency of control cells was 30.9% throughout the experimental period. The mean lethal dose ($D_0$) was measured from the straight portion of the survival curve as the dose required to reduce survival to 37%, and the extrapolation number ($n$) was measured by extrapolation of the straight portion of the survival curve to the ordinate. The two parameters for each survival curve were determined from least squares linear regression.

Dose-response survival curves of HMV-II melanoma cells after one hour's treatment with each drug are shown in Figure 2, and the $D_0$ and $n$ values of each drug are summarized in Table I. The $D_0$ value of L-dopa, examined for the purpose of comparison, was $3.16 mg ml^{-1}$, and L-dopa was demonstrated to be considerably weak in killing melanoma cells. On the contrary, 4-S-cysteinylcatechol, a sulphur homologue of L-dopa, had a $D_0$ value of $0.01 mg ml^{-1}$, and showed remarkably potent killing effects

**Figure 1** Chemical structures of the synthetic compounds and L-dopa. $R = -CH_2CH(NH_2)COOH$.

Correspondence: I. Yamada.
Received 26 June 1988.

**Figure 2** Effects of the drugs on the survival of HMV-II human melanoma cells. The cells were exposed to the different concentrations of each drug for 1 h at 37°C. The cell survival was estimated by the ability to form viable colonies, and the results are expressed as a fraction of control cell survival. Values represent mean of 3 to 5 determinations. All standard deviations were less than 10%. ●, 4-S-CC; □, 2-S-CH; ▲, 3-S-C-5-MC; △, 3-S-CC; ■, 2-S-CR; ○, L-dopa.

**Table I** Killing effects of 4-S-CC and related analogs on HMV-II human melanoma cells

| *Drug* | *Parameters*[a] $D_0$ (*mg ml*$^{-1}$) | *n* | $D_0$ *ratio* (*L-dopa/drug*) | *Significance* (*P value*)[b] |
|---|---|---|---|---|
| 4-S-CC | 0.010 | 1.0 | 316.00 | <0.01 |
| 2-S-CH | 0.035 | 2.1 | 90.29 | <0.01 |
| 3-S-C-5-MC | 0.271 | 1.2 | 11.66 | <0.01 |
| 3-S-CC | 1.68 | 1.0 | 1.88 | NS[c] |
| 2-S-CR | 6.02 | 1.0 | 0.52 | NS |
| L-dopa | 3.16 | 0.9 | 1.00 | – |

[a]Each $D_0$ and *n* value is an average from 3 to 5 separate experiments; [b]The significance was calculated between L-dopa and other drugs by the Student's *t* test; [c]NS, not significant.

on melanoma cells compared with L-dopa. When the effects of the two drugs were compared on the basis of the ratio of the $D_0$ value, 4-S-CC was ~316-fold more potent in killing melanoma cells than L-dopa ($P<0.01$). 3-S-Cysteinylcatechol had a $D_0$ value of 1.68 mg ml$^{-1}$; the killing activity of 3-S-CC was comparable to L-dopa in potency. However, 3-S-cysteinyl-5-methylcatechol, a methyl conjugate of 3-S-CC, had a $D_0$ value of 0.271 mg ml$^{-1}$, and was intermediate among the drugs examined (Table I). The $D_0$ ratio of 3-S-C-5-MC in reference to L-dopa was 11.66 ($P<0.01$). 2-S-Cysteinylhydroquinone had a $D_0$ value of 0.035 mg ml$^{-1}$ and was remarkably potent in killing melanoma cells. The killing activity of 2-S-CH was next to that of 4-S-CC in potency, and the $D_0$ ratio in reference to L-dopa was 90 ($P<0.01$). However, 2-S-cysteinylresorcinol had a $D_0$ value of 6.02 mg ml$^{-1}$ and was the least effective of the drugs examined.

Our results demonstrate that 4-S-cysteinylcatechol, a sulphur homologue of L-dopa, is a remarkably potent agent in killing human melanoma cells as compared with L-dopa itself. The enhanced toxicity of 4-S-CC to melanoma cells may be related to the presence of a sulphur atom in the molecule, the only difference between 4-S-CC and L-dopa in chemical structure (Figure 1). Ito *et al.* (1987) have indicated that 4-S-CC is a much better substrate for tyrosinase than L-dopa, suggesting that this may be due to the electron donating resonance effect of the sulphur atom. L-dopa and its analogues have been shown to be selectively cytotoxic to melanoma cells (Wick *et al.*, 1977; Wick, 1978), and its mechanism has been postulated to be tyrosinase-mediated oxidation of these compounds to *o*-quinone forms that inhibit the activity of DNA polymerase α (Graham *et al.*, 1978; Wick, 1980). Thus, the enhanced toxicity of 4-S-CC may be ascribed to a significantly increased affinity for tyrosinase. In addition, the sulphur atom may also increase the incorporation of these compounds into cells by virtue of its lipophilicity (Ito *et al.*, 1981).

2-S-Cysteinylhydroquinone was next to 4-S-CC in killing melanoma cells. Hydroquinone itself has been shown to have melanocytolytic activity against melanocytes *in vivo* (Jimbow *et al.*, 1974), and Chavin *et al.* (1980) showed that hydroquinone significantly prolongs the survival of melanoma-bearing mice. Recently, Penney *et al.* (1984) suggested that the cytotoxicity of hydroquinone to melanoma cells may be via its oxidation by tyrosinase. Thus, the potent killing activity of 2-S-CH may also be related to the tyrosinase-mediated oxidation of the hydroquinone moiety.

3-S-Cysteinyl-5-methylcatechol had an intermediate activity in killing melanoma cells among the drugs examined. Since Fujita *et al.* (1980) have demonstrated the 5-S-cysteinyldopa, an intermediate in the pathway from L-dopa to pheomelanin, is much more cytotoxic to melanoma cells than L-dopa, the enhanced effect of 3-S-C-5-MC may be related to the fact that its chemical structure is closely analogous to that of 5-S-cysteinyldopa (Figure 1).

Our results indicate that 4-S-CC and its analogues possess remarkably potent killing activities to human melanoma cells, and that these effects are closely related to their chemical structure. L-dopa was found to have only a weak killing effect, though it has been reported that L-dopa inhibits selectively the growth of melanoma cells (Wick *et al.*, 1977). Thus, 4-S-CC and analogues may make a new, effective cytotoxic agent for the rational chemotherapy that can ameliorate malignant melanoma.

### References

CHAVIN, W., JELONEK, E.J. JR., REED, A.H. & BINDER, L.R. (1980). Survival of mice receiving melanoma transplants is promoted by hydroquinone. *Science*, **208**, 408.

FUJITA, K., ITO, S., INOUE, S. & 4 others (1980). Selective toxicity of 5-S-cysteinyldopa, a melanin precursor, to tumor cells *in vitro* and *in vivo*. *Cancer Res.*, **40**, 2543.

GRAHAM, D.G., TIFFANY, S.M. & VOGEL, F.S. (1978). The toxicity of melanin precursors. *J. Invest. Dermatol.*, **70**, 113.

ITO, S., INOUE, S., YAMAMOTO, Y & FUJITA, K. (1981). Synthesis and antitumor activities of cysteinyl-3,4-dihydroxyphenylalanines and related compounds. *J. Med. Chem.*, **24**, 673.

ITO, S., KATO, T., ISHIKAWA, K., KASUGA, T. & JIMBOW, K. (1987). Mechanism of selective toxicity of 4-S-cysteinylphenol and 4-S-cysteaminylphenol to melanocytes. *Biochem. Pharmacol.*, **36**, 2007.

JIMBOW, K., OBATA, H., PATHAK, M.A. & FITZPATRICK, T.B. (1974). Mechanism of depigmentation by hydroquinone. *J. Invest. Dermatol.*, **62**, 436.

KASUGA, T., FURUSE, T., TAKAHASHI, I. & TSUCHIYA, E. (1971). Ultrastructural and autoradiographic studies on melanin synthesis and membrane system using cultured B16 melanoma, irradiated melanoma, and human malignant melanoma. In *Biology of Normal and Abnormal Melanocytes*, Kawamura, T. *et al.* (eds) p. 241. University of Tokyo Press: Tokyo.

PAWELEK, J.M. (1976). Factors regulating growth and pigmentation of melanoma cells. *J. Invest. Dermatol.*, **66**, 201.

PENNEY, K.B., SMITH, C.J. & ALLEN, J.C. (1984). Depigmenting action of hydroquinone depends on disruption of fundamental cell processes. *J. Invest. Dermatol.*, **82**, 308.

WICK, M.M. (1978). Dopamine: A novel antitumor agent against B-16 melanoma *in vivo*. *J. Invest. Dermatol.*, **71**, 163.

WICK, M.M. (1980). Levodopa and dopamine analogs as DNA polymerase inhibitors and antitumor agents in human melanoma. *Cancer Res.*, **40**, 1414.

WICK, M.M., BYERS, L. & FREI, E., III (1977). L-dopa: Selective toxicity for melanoma cells *in vitro*. *Science*, **197**, 468.